# Links between pet ownership and exercise on the mental health of veterinary professionals

**DOI:** 10.1002/vro2.62

**Published:** 2023-05-23

**Authors:** Elliot T. Smith, Ana Maria Barcelos, Daniel S. Mills

**Affiliations:** ^1^ Department of Life Sciences University of Lincoln Lincoln Lincolnshire UK

## Abstract

**Background:**

Connections between the effects of pet ownership and exercise on mental health have previously been demonstrated in different populations. However, little is known about the potential effects of pet ownership and exercise on the mental health of veterinary professionals. Since these individuals have a high prevalence of poor mental health and suicide, while they deal with pets professionally, we investigated the impact of pet ownership, exercise and different types of pet ownership on this demographic group.

**Method:**

Veterinary professionals over 18 years old answered an online questionnaire about pet ownership, exercise, mental health (including anxiety, depression and suicidal ideation) and mental health correlates. Regression models were used to identify variables significantly related to mental health outcomes.

**Results:**

Of 1087 respondents, pet owners were more depressed than non‐owners, while anxiety or suicidal ideation was not associated with pet ownership. Dog and horse owners were psychologically healthier (less anxiety, less suicidal ideation) than non‐owners of these species. Veterinary professionals who ran regularly had lower anxiety and depression. Those who walked regularly and spent less time sitting experienced fewer depression symptoms.

**Conclusions:**

Running, walking and avoiding prolonged sitting might protect the mental health of veterinary professionals. The type of pet owned may be an important factor in the relationship between pet ownership and mental health; however, generally, pet ownership was associated with worse mental health outcomes in this demographic group. Future studies should determine the causal nature of these relationships.

## INTRODUCTION

Poor mental health within the veterinary profession has been a concern for many years.^1^ The proportional mortality ratio for suicide (PMR, proportion of deaths due to suicide in relation to the total deaths in a given time period in a specified population) in UK veterinarians is approximately four times that of the general population and twice that of other healthcare professionals.^2^ The problem is not restricted to the UK, as high levels of suicide have been reported among veterinarians in Australia, USA and Norway.^3^, ^4^, ^5^

Suicide has multiple risk factors, including anxiety and depression.^6^, ^7^ The prevalence of anxiety (26.3%) and depression (5.8%) previously reported in the UK veterinary profession is significantly higher than that in the general population.^8^ It therefore seems reasonable to suggest that poor mental health among veterinarians may contribute to the high suicide rate within the profession.^8^

In a UK survey of 6988 veterinarians, almost 90% of them indicated that their work was stressful.^9^ Elevated work stress can precipitate anxiety and depression,^10^, ^11^ which contribute to suicide risk.^12^, ^13^ Particular concerns may include long working hours, frequency of performing euthanasia,^14^ poor work–life balance and potentially conflict over care priorities with owners.^5^, ^15^ Female veterinarians in the early stages of their career are more likely to suffer from stress and depression^16^ and have a higher PMR for suicide than males.^1^ Given that a greater proportion of females are entering the profession and the high prevalence of suicidal ideation among veterinarians (21.3% in a 12‐month period),^8^ more effort is required to improve wellbeing in the profession.

Pet ownership and exercise are both commonly reported moderators of positive human wellbeing in the general population.^17^, ^18^, ^19^, ^20^, ^21^, ^22^ Pets can enhance social interactions, alleviate loneliness and directly improve wellbeing through the relationship and emotional support they provide.^18^ They can act as a buffer during times of stress, protect against anxiety‐related illness and may alleviate depressive symptoms.^18^, ^23^, ^24^ It is therefore no surprise that pets have been associated with improvements in certain mental health conditions.^25^, ^26^, ^27^ However, studies of the impact of pet ownership on wellbeing are inconsistent, several reporting indifferent or negative effects related to pet ownership on mental and physical health,^28^, ^29^, ^30^ with higher levels of pet attachment often associated with depression.^31^, ^32^, ^33^

Evidence that physical activity has beneficial effects on mental health is supported by extensive reports of reduced anxiety and depression associated with increased physical activity and exercise.^19^ ^−^ ^22^ Subsequent noradrenergic and opioid activation from exercise has been linked to antidepressant effects and mood enhancement respectively.^22^ While there was no significant difference in health‐related quality of life between veterinary students who met physical activity guidelines and veterinary students who did not,^34^ a potential link between physical activity and wellbeing has yet to be explored in veterinary professionals.

To date, studies assessing mental health within the veterinary profession have focused on veterinarians. Reports have indicated high levels of occupational stress, burnout and poor mental health in veterinary technicians, nurses and support staff,^35^, ^36^, ^37^ with an elevated risk of suicide among veterinary nurses.^38^

To the best of the authors’ knowledge, no study has assessed the impact of pet ownership and exercise on mental health across the spectrum of veterinary professionals. This cohort is likely to differ from the general population given the prediction that a greater number of this population are likely to be pet owners and also meet the physical activity guideline due to the physical nature of the work and lifestyle associated with pet ownership.

Veterinary professionals were asked to respond to an online questionnaire, which allowed us to answer four research questions. First, was there a significant difference in self‐reported depression, anxiety and suicidal ideation among pet owning and non‐pet owning veterinary professionals? Second, was the species owned related to these mental health indicators? Third, was the level of physical activity performed by veterinary professionals associated with depression, anxiety or suicidal ideation? Fourth, was the performance of a particular type of exercise and time spent sitting associated with better outcomes in these mental health indicators?

## METHODS

### Survey development and content

A 10‐min online survey (SQ1) was developed to address the four aims of the study. Piloting was undertaken with eight veterinary professionals known to the author. These professionals were not eligible to participate in the final survey. A priori power analysis was calculated in G*Power. *F* tests were chosen as the test family; multiple linear regression was selected as the statistical test. Power was set at 80%, alpha at 0.05 and the number of predictors at around 30. A small to medium effect size (*f* ^2^ = 0.07) was chosen. With these parameters, a minimum sample size of 374 was obtained.

Symptoms of anxiety and depression were assessed with validated and internationally used instruments, the seven‐item Generalised Anxiety Disorder scale (GAD‐7)^39^ and the nine‐item Patient Health Questionnaire (PHQ‐9),^40^ respectively. In the GAD‐7, a score greater than 10 is indicative of generalised anxiety disorder, with 15–21 representing severe anxiety, 10–14 moderate anxiety and 5–9 mild anxiety.^39^ On the PHQ‐9 scale, a score of 20 or more indicates severe depression, 15–19 moderately severe depression, 10–14 moderate depression, 5–9 mild depression and 0–4 minimal depression.^41^ Suicidal ideation was assessed using a customised item selected from a questionnaire developed in another study^40^: ‘In the previous 12 months have you thought of taking your life, even if you would not really do it?’.  The choice of this question to assess suicidal ideation enabled comparison with a study^8^ that used this item in an assessment of the mental health of veterinarians in the UK. Due to the personal nature of the questions involved in the survey, participants were provided with contact numbers for Samaritans and Vetlife for support on their mental health.

To assess pet ownership status, participants were asked to indicate if they currently owned a pet and specify what kind of pet(s), including dog, cat, rabbit, reptile, horse, fish, rodent, invertebrates and other. Due to low frequency, rabbits, reptiles, rodents and invertebrates were merged with ‘other’ in the statistical analysis. Pet ownership status and each pet type were dichotomised in the regression models (yes/no).

The level of physical activity performed by the veterinary professionals was measured using the International Physical Activity Questionnaire (IPAQ).^42^ The IPAQ was chosen due to its brevity (seven items), validity and international usage. It assesses the duration and frequency of physical activities over the previous 7 days. According to the IPAQ scoring protocol,^43^ the total physical activity score of participants is categorised into high, moderate or low levels. The moderate IPAQ category equates to at least 30 min of moderate physical activity on most days, which meets the recommended UK physical activity guideline of at least 150 min of moderate to vigorous exercise per week.^44^ In addition, a question was developed to assess regular involvement of participants in different forms of exercise, including gym, running, swimming, horse riding, cycling, walking/hiking, court‐related sports, other and none. Each of these forms of exercise was used as a dichotomous variable (yes/no) in the regression models.

The survey assessed participants' age, gender, country of residence, relationship status, whether they lived with other people and if they were a parent. Participants also provided information about their employment status, position within the veterinary profession, veterinary sector and the proportion of time they spent working with animals. For those who owned pets, the reason for pet acquisition was also requested. Each of these questions was treated as a categorical variable with two or more levels in the regression models. Although they were not a main focus of the study, they were accounted for as covariates due to the lack of information on these matters and the possibility of them influencing the dependent variables (depression, anxiety and suicidal ideation).

### Participants

A flyer advertising recruitment for veterinary professionals to take part in a survey on their mental health was disseminated on social media platforms. Groups were targeted on Facebook pages, including Veterinary Voices UK and Vets Stay Go Diversify, together with Reddit, WhatsApp and Twitter, over a 4‐week period during June and July 2021. Links to the survey (including a QR code) were provided on the flyer. Convenience sampling was used. Participation was voluntary and no incentives were offered. To avoid biasing the sample towards veterinary professionals who own pets or perform physical activities, the study aims were only disclosed to participants after survey completion. Participants were asked to participate to provide a greater understanding of mental health within the profession due to concerns related to poor mental health in this cohort. Informed consent was obtained prior to commencing the survey.

Self‐reported veterinary professionals from any location who were currently working or used to work in the profession were eligible for participation in the study. Veterinary professionals under 18 years old were automatically directed to the end of the survey and were not included in the data analysis because the minimal age for participation was 18 years old. To ensure consistency and full completion of the survey, each question required completion before the participant was enabled to proceed to the subsequent question.

### Statistical analysis

Data were initially assessed for normality and all analyses were performed using R version 4.0.3 (R Core Team; R: a language and environment for statistical computing. Vienna, Austria: R Foundation for Statistical Computing; 2020).

Two general linear models, one predicting depression severity (PHQ‐9 score) and the other anxiety (GAD‐7 score), were fitted to test whether pet ownership status, level of physical activity and type of exercise were associated with these mental health issues (*n* = 1088). The PHQ‐9 and GAD‐7 scores were square root transformed to meet the assumptions of the test. Suicidal ideation was dichotomous (yes/no) and a generalised linear model for binary outcomes was used to test the same potential associations as per depression and anxiety.

The homogeneity of variances across the groups of categorical variables was tested using Levene's test (see Supporting Information S1). Type I ANOVA (with sequential sum of squares) was used to identify the significant variables.

In the pet ownership models (*n* = 1088), the variable pet ownership was placed first in the list of variables followed by the covariates (except ‘reason for pet acquisition’) as described in the section ‘Survey development and content’. The covariates were included in these models to account for their potential influence on the dependent variables. These covariates included participants' age, gender, country of residence, relationship status, whether they lived with other people, whether they were a parent, their employment status, position within the veterinary profession, their veterinary work sector and proportion of time spent working with animals. To test whether pet type was significantly associated with depression, anxiety and suicidal ideation, each of the three models described above was repeated with participants who only owned pets (*n* = 941), with the variable ‘reason for pet acquisition’ added as an additional covariate to the models.

*F* tests were used to calculate *p*‐values for each of the models described above. Backward stepwise elimination procedures were performed (where the least statistically significant variables were discarded one by one, based on the *F* test results) to produce the minimum adequate model with reduced degrees of freedom and the risk of spurious associations. Post hoc Tukey tests were performed on significant categorical variables with three or more levels from the general linear models to identify which were significantly different. Chi‐squared tests were performed on significant categorical variables with three or more levels for the generalised linear models to assess the significant levels based on adjusted residuals greater than |1.96|. Descriptive statistics (mean, SD, median, interquartile range [IQR]) and percentages were used to describe the data. Statistical significance was defined as a *p*‐value less than 0.05. A more conservative significance threshold was not chosen to avoid missing potentially important associations.

To illustrate the estimated parameters of the models and their standard error/confidence intervals, the final models (with significant variables only) were tested with the summary () function in R. These complementary results are shown in Tables S8–S13. Although the significant variables from the summary function were very similar to the ones identified in the ANOVA tests described in the paper, they were not identical because type I ANOVA takes into consideration the order of the variables, whereas to generate the estimate parameters, the order of the variables is disregarded.

## RESULTS

### Demographics and characteristics of participants

A total of 1088 veterinary professionals completed the survey (Table 1). One participant did not meet the age criteria and was thus excluded from the study. Over 80% of participants were residents in the UK and most people were in full or part‐time employment (89.1%). The 31–40 age group was most prevalent (39.3%); females accounted for 86.9% of participants, which is more female biased than the general population working in the veterinary profession.^45^ Veterinarians represented over half of the veterinary professionals (62.7%) and small animal first opinion was the most common sector of work (68.6%). Only two participants reported that they were no longer working in the veterinary profession. Most participants spent more than 75% of time working with animals.

**TABLE 1:** Demographics and characteristics of participants (*n* = 1087).

Category	*n*	%
**Veterinary professional**		
Veterinary surgeon	682	62.7
Veterinary nurse	188	17.3
Non‐clinical position	136	12.5
Technician	58	5.3
Other	23	2.1
**Place of residence**		
UK	884	81.3
North America	104	9.6
Other	99	9.1
**Age (years)**		
18–30	375	34.5
31–40	427	39.3
41–50	175	16.1
51 and over	110	10.1
**Gender**		
Female	945	86.9
Male	130	12.0
Identify in another way	12	1.1
**Relationship status**		
In a relationship	701	64.5
Not in a relationship	232	21.3
Dating	154	14.2
**Living with people**		
Yes	889	81.8
No	198	18.2
**Parent**		
Yes	385	35.4
No	702	64.6
**Employment status**		
Full/part time	1039	95.6
Unemployed	48	4.4
**Veterinary sector** ^a^		
Small animal first opinion	760	69.9
Equine first opinion	209	19.2
Farm first opinion	125	11.5
Referral small animal	113	10.4
Referral equine	80	7.4
Industry	20	1.8
Education, public sector, research	88	8.1
Other	51	4.7
**Proportion of time working with animals**		
0%	101	9.3
25%	100	9.2
50%	107	9.8
75%	445	40.9
100%	334	30.7
**Pet ownership**		
Pet owner	941	86.6
Non‐pet owner	146	13.4
**Type of pet owned** ^a^		
Dog	688	73.0
Cat	514	54.5
Horse	172	18.2
Fish	108	11.5
Other	289	16.3
**Reason for pet ownership**		
Companionship	743	79.0
Exercise	107	11.4
Other	91	9.7
**International Physical Activity Questionnaire (physical activity category)**		
High physical activity	416	38.2
Moderate physical activity	446	41.0
Low physical activity	225	20.7
**Generalised Anxiety Disorder‐7 (anxiety)**		
Minimal	357	32.8
Mild	319	29.3
Moderate	234	21.5
Severe	177	16.3
**Patient Health Questionnaire‐9 (depression)**		
Minimal	365	33.6
Mild	295	27.1
Moderate	221	20.3
Moderate/severe	130	12.0
Severe	76	7.0
**Suicidal ideation**		
No	715	65.8
Yes	372	34.2
**Exercise** ^a^		
Gym	264	24.3
Running	261	24.0
Sports	88	8.1
Swimming	137	12.6
Horse riding	187	17.2
Cycling	180	16.6
Walking	792	72.9
Other	104	9.6
No exercise	110	10.1

^a^ Percent exceeds 100% in these categories as participants were able to select multiple choices.

Over 86% were pet owners (*n* = 941) and dogs were the most popular owned pet, with companionship being the main reason for pet acquisition. Almost 80% of participants met physical activity guidelines partaking in moderate or high physical activity. Walking was the most popular form of exercise (*n* = 792, 72.9%), followed by going to the gym (*n* = 264, 24.3%) and running (*n* = 261, 24.0%). The mean total physical activity score was 2264.4 MET (MET stands for the metabolic equivalent of task, it is used to indicate energy expenditure)/min/week (SD = 2079.5, median = 1706, IQR = 2232.5); and the mean time spent sedentary was 5.2 h/day (SD = 3.4, median = 4.0, IQR = 3.0). Further participant details and descriptive statistics of psychological health measures for each characteristic are included in Tables 1 and S1.

In the following sections, the results of the regression models used to answer the research questions of this study are described. The use of comparative adjectives (such as higher, lower) in these sections implies that the respective variable was significant (*p* < 0.05) in the regression model it is referred to.

### Is pet ownership associated with anxiety, depression and suicidal ideation in the profession?

Descriptive level data relating to the relationship between whether a respondent owned a pet and the mental health outcomes (anxiety, depression and suicidal ideation) are summarised in Table 2. Only depression scores significantly differed between the two pet ownership groups. Pet owners experienced higher depression than non‐pet owners (*F*₁_,_₁₀₆₄  = 7.19, *p* = 0.007, Table S2). Details of the models assessing the impact of pet ownership on mental health (*n* = 1087) are provided in Tables S2–S4.

**TABLE 2:** Descriptive summary scores of Generalised Anxiety Disorder (GAD‐7), Patient Health Questionnaire (PHQ‐9) and reported suicidal ideation for pet ownership.

Characteristic	GAD‐7 score mean (Standard deviation)	PHQ‐9 score mean (SD)	Suicide ideation (%)
Pet owner (*n* = 941)	8.3 (5.7)	8.8 (6.4)	34.0
Non‐pet owner (*n* = 146)	7.5 (5.5)	7.4 (6.2)	32.2
Overall (*n* = 1087)	8.2 (5.7)	8.6 (6.4)	34.2

*Note*: GAD‐7 scores, 5–9: mild anxiety and ≥10: generalised anxiety disorder. PHQ‐9 scores, 5–9: mild depression and 10–14: moderate depression.

### Is exercise associated with anxiety, depression and suicidal ideation in the profession?

Level of physical activity was only associated with depression (*F*_2,1064_ = 3.59, *p* = 0.028). Those who partake in moderate physical activity showed fewer symptoms of depression compared to those who engage in low levels of activity (post hoc Tukey test, *p* = 0.030), while no difference was found between high activity level and the others.

Runners had significantly reduced anxiety (*F*_1,1064_ = 15.56, *p* < 0.001) and depression (*F*_1,1064_ = 16.87, *p* < 0.001) compared to non‐runners. Walking as a form of exercise was also associated with lower depression (*F*_1,1064_ = 3.86, *p* = 0.049) compared to not walking. On the other hand, increased time sitting was associated with higher depression scores (*F*_1,1064_ = 4.95, *p* = 0.026).

Further statistical details about exercise and mental health (*n* = 1087) are available in Tables 3 and S2–S4.

**TABLE 3:** Descriptive summary scores of Generalised Anxiety Disorder (GAD‐7), Patient Health Questionnaire (PHQ‐9) and reported suicidal ideation for exercise.

Variable	GAD‐7 score, mean (Standard deviation)	PHQ‐9 score, mean (SD)	Suicidal ideation (%)
Running			
Yes (*n* = 261)	6.8 (5.0)	30.3	30.3
No (*n* = 826)	9.2 (6.6)	35.5	35.5
Walking			
Yes (*n* = 792)	8.0 (5.6)	8.3 (6.2)	32.8
No (*n* = 295)	8.7 (5.9)	9.5 (6.7)	38.0
Gym			
Yes (*n* = 264)	8.3 (5.7)	8.3 (6.4)	36.7
No (*n* = 823)	8.2 (5.7)	8.7 (6.4)	33.4
International Physical Activity Questionnaire category			
High activity (*n* = 416)	8.3 (5.9)	8.5 (6.5)	31.2
Moderate activity (*n* = 446)	7.8 (5.4)	8.2 (6.1)	35.4
Low activity (*n* = 225)	8.7 (5.6)	9.7 (6.5)	37.3

### Is type of pet owned associated with anxiety, depression and suicidal ideation in the profession?

The results described in this section refer to the 941 pet owning veterinary professionals. The remaining participants did not own a pet and therefore could not be included in these analyses. Dog owners had significantly lower anxiety scores (*F*₁_,918_ = 5.56, *p* = 0.019) and suicidal ideation (*χ* ^2^(1) = 5.04, *p* = 0.025) than non‐dog owners. Similarly, horse owners were psychologically healthier than non‐horse owners in all mental health indicators assessed: anxiety (*F*_1,918_ = 5.60, *p* = 0.018), depression (*F*_1,920_ = 8.34, *p* = 0.004) and suicidal ideation (*χ* ^2^(1) = 4.32, *p* = 0.038). In contrast, veterinarian professionals who owned a cat experienced more depression (*F*_1,920_ = 4.37, *p* = 0.037) and suicidal ideation (*χ* ^2^(1) = 10.52, *p* = 0.001) than non‐cat owners. Likewise, owners of ‘other’ pets also had poorer mental health with more anxiety (*F*_1,918_ = 12.52, *p* < 0.001) and depression (*F*₁_,_₉₂₀ = 5.92, *p* = 0.015). Finally, fish ownership was not associated with any of the mental health indicators. Pet owners who obtained pets for exercise purposes had lower anxiety than those who acquired pets for companionship (*F*_2,918_ = 3.50, *p* = 0.031). Table 4 summarises the mental health outcomes in veterinary professionals who owned a pet across the different types of pet owned. The regression models on pet type and mental health outcomes are reported in Tables S5–S7.

**TABLE 4:** Descriptive summary scores of Generalised Anxiety Disorder (GAD‐7), Patient Health Questionnaire (PHQ‐9) and reported suicidal ideation among veterinary professionals for type of pet owned.

Pet type	GAD‐7 score, mean (Standard deviation)	PHQ‐9 score, mean (SD)	Suicidal ideation (%)
Dog owner			
Yes (*n* = 688)	8.1 (5.8)	8.6 (6.4)	34.5
No (*n* = 253)	8.8 (5.5)	9.2 (6.5)	40.3
Cat owner			
Yes (*n* = 514)	8.7 (5.7)	9.2 (6.6)	39.9
No (*n* = 427)	7.9 (5.7)	8.3 (6.1)	28.1
Horse owner			
Yes (*n* = 172)	7.3 (5.6)	7.7 (6.1)	26.7
No (*n* = 759)	8.5 (5.7)	9.1 (6.4)	36.8
Fish owner			
Yes (*n* = 108)	8.4 (5.6)	9.2 (6.4)	38.0
No (*n* = 833)	8.3 (5.7)	8.7 (6.4)	34.1
Other pets			
Yes (*n* = 289)	9.2 (5.6)	9.6 (6.3)	30.8
No (*n* = 652)	8.0 (5.7)	8.5 (6.4)	36.2

*Note*: GAD‐7 scores, 5–9: mild anxiety and ≥10: generalised anxiety disorder. PHQ‐9 scores, 5–9: mild depression and 10–14: moderate depression.

### Other factors linked with anxiety, depression and suicidal ideation in the profession

Although this study did not aim to investigate the impact of the covariates (including age, gender, country) on mental health, a summary of the significant covariates in the models of anxiety, depression and suicidal ideation with the whole sample (*n* = 1087) is provided, as this might be useful for future investigations in the field (Table 5). Full details of these models are available in Tables S2–S4.

**TABLE 5:** Summary of covariates and their significance in models used to assess the impact of pet ownership status and exercise on mental health of veterinary professionals (*n* = 1087).

Variable	Significance of variable within model		
	Anxiety	Depression	Suicidal ideation
Pet ownership		++	
Age	***	***	***
Gender	***	**	
Country		*	
Relationship status		**	**
Living with people			–
Parent			– –
Employment status (being employed)			+
Profession within the field	***	***	
Small animal first opinion sector	+++	+++	+++
Equine first opinion sector	–	–	–
Farm first opinion sector	– – –	– – –	
Referral small animal sector			+++
Proportion of time working with animals	+		
Physical activity category		*	
Running	– – –	– – –	
Walking		–	
Time sitting		+	

*Note*: Empty cell—not significant in the model. */+/– indicates *p* < 0.05; **/++/– – indicates *p* < 0.01; ***/+++/– – – indicates *p* < 0.001. Symbol ‘+’ indicates a positive association between variables and symbol ‘–’ indicates a negative association.

## DISCUSSION

Our findings suggest that pet owning veterinary professionals have significantly higher depression compared to non‐pet owning professionals. However, no difference was found in anxiety or suicidal ideation between these groups (Tables 2 and 5). The type of pet owned was significantly related to mental health indicators among pet owning professionals. Horse and dog owners were apparently psychologically healthier than those who did not own these animals, while owners of cats and ‘other pets’ potentially had worse mental health than non‐cat/‘other pets’ owners (Table 4). Although high level of physical activity was not associated with better mental health outcomes than lower levels of physical activity, it was found that running and walking may be protective factors, while prolonged sitting might be a risk factor for poorer mental health within the profession (Table 3).

Other studies have similarly reported associations between pets and increased risk of depression.^30^, ^46^ Another investigation found no difference in depression between older adult pet owners and non‐pet owners.^29^ On the other hand, there are reports that support ownership of pets as an adjunct to treat depression, especially in those members of the general population who are fond of animals, with suggestions that pets can counteract anhedonia by providing responsibility and promoting physical activity.^25^

Given that veterinary professionals spend a large proportion of their time working with animals, it is possible that they do not experience the same advantages from owning a pet as other cohorts. To explore this hypothesis further, studies could be conducted with different professions that work with animals. It is also conceivable that veterinary professionals may acquire pets in the hope that it may alleviate depression and that the benefits of pets might only come into play when the person is experiencing highly stressful or adverse events,^47^ which might not be captured in a cross‐sectional investigation such as this. Finally, it is possible that veterinary professionals are more likely to adopt a pet with behaviour problems or physical health issues that may present additional stress/burden in their lives, worsening their mental health.^48^, ^49^

Other studies have reported that dog owners are more likely to meet physical activity guidelines^50^ and exercise more than owners of other pets,^51^ which can consequently improve mental health.^52^ Comparable findings in horse owners may also explain their better mental health.^53^ Furthermore, people who obtained pets for exercise purposes had lower anxiety than those who acquired pets for companionship. Pet type could also influence the type of activities and interactions shared with the pet, which in turn can benefit wellbeing.^48^ For example, engagement in more frequent social interactions and activities may well occur through ownership of a dog^54^ and horse,^55^ compared to cats and other pets. The level of emotional closeness to dogs has also been found to provide greater benefits from dog‐related activities, in turn improving mental wellbeing.^56^

Personality traits have been recognised to predispose vulnerability to poor mental health.^57^ Previous studies comparing dog and cat owners have recognised differences in personality traits between these owners.^58^ Dog owners have traits of being more agreeable, extraverted and less neurotic than cat owners, which may account for better wellbeing among dog owners.^58^ It is therefore possible that personality traits influence choice of pet type,^59^ and that it is personality rather than pet type that affects vulnerability to poor mental health. Future studies should investigate personality traits of different types of pet owners within the veterinary profession.

Despite speculation that pet ownership can have protective effects against suicide,^60^ previous studies have found no significant relationship.^61^, ^62^ However, our results support proposals that pets, particularly dogs and horses, can have a protective role, although it cannot be generalised to all pets given that cat owners experienced significantly higher suicidal thoughts. If indeed there is a causal association between pet type and suicidal ideation, it might be that pet‐related risk factors for suicidal ideation (such as pet behavioural problems and pet health issues) are more negatively perceived by cat owners, or that pet‐related protective factors against suicide (such as companionship^63^, ^64^) are less evident in this species.^26^

Eighty percent of the veterinary professionals in this study met minimum physical activity guidelines. Increased physical activity is generally associated with reduced anxiety and depression.^20^ However, veterinary professionals performing high levels of physical activity did not differ from those performing low or moderate levels of physical activity (Table 3). Increased activity in veterinary professionals might not have the same impact as in the general population because a large proportion of the working day is spent active. There was still evidence to support the benefits of physical activity on mental health, particularly from running, walking and spending less time sitting, which correlates with extensive evidence of this relationship.^65^

The cross‐sectional nature of the study does not allow causal relationships to be determined. Our recruitment strategy will have biased the sample towards veterinarians. Nonetheless, this work highlights the need to attend to the mental health of those working alongside veterinarians. The self‐reported measures of mental health and physical activity used in our survey could potentially lead to overestimated results. While recruitment may have inadvertently attracted an audience with poorer mental health, social media groups were not specifically targeted at groups with poor mental health. Three‐quarters of participants were under the age of 40 years, which is greater than previous studies, and this could reflect the nature of recruitment through social media, which may attract a younger audience. Therefore, generalisability of results within the veterinary profession may be impaired. A complication within a study such as this is the non‐mutually exclusive nature of pet ownership, where respondents may own several types of pets. This prevents comparison of one type of pet ownership versus another. However, exclusion of participants with multiple pet species would produce an unrepresentative sample of the population. Finally, despite cessation of the COVID‐19 national lockdown in the UK prior to study commencement, the knock‐on effects could explain some of the high levels of depression and anxiety symptoms reported here.

This study has identified an association between pet ownership and poor mental health within the veterinary profession, with those who owned pets reporting more symptoms of depression than non‐owners. In addition, the type of pet owned could play an important role. Our work also highlights the value of certain forms of aerobic exercise on mental health. However, higher levels of general physical activity were not necessarily associated with better mental health. Studies that allow the determination of causal relationships between pet ownership/pet type/exercise and mental health indicators in veterinary professionals are needed and these should control for the significant covariates identified.

## AUTHOR CONTRIBUTIONS

Elliot T. Smith designed the study, collected and analysed the data and wrote the first draft of the article. Ana Maria Barcelos and Daniel S. Mills designed the study, revised the data analysis and wrote the article.

## CONFLICTS OF INTEREST STATEMENT

The authors declare they have no conflicts of interest.

## FUNDING INFORMATION

The authors received no specific funding for this work.

## ETHICS STATEMENT

This study was approved by the ethics committee of the University of Lincoln (reference 2021_1069) and conformed to the Declaration of Helsinki.

## Supporting information

Supporting Information

## Data Availability

Data available upon reasonable use request from the authors.
