# Supplementary material for: Links between pet ownership and exercise on the mental health of veterinary professionals
Source: Vet Rec Open. 2023 May 23;10(1):e62. doi: 10.1002/vro2.62 (PMC10206414; doi:10.1002/vro2.62)
Supplement: Supplementary file 1 — Supporting Information [file VRO2-10-e62-s001.pdf]

Supporting Information

Table S1 Central tendency and spread of GAD-7 (Generalised Anxiety Disorder scale) and PHQ-9 scores (Patient Health Questionnaire); proportion of suicidal ideation for demographics and characteristics (n = 1087)

Characteristic	GAD-7 score mean (standard deviation); median (Interquartile range)	PHQ-9 score Mean (SD); median (IQR)	Suicidal ideation %
**Age**			
18 – 30 (n = 375)	9.5 (5.5); 9.0 (8.0)	10.1 (6.5); 9.0 (9.5)	42.9
31 – 40 (n = 427)	7.9 (5.5); 7.0 (8.5)	8.2 (6.2); 7.0 (9.0)	30.9
41 – 50 (n = 175)	7.9 (5.8); 6.0 (9.0)	7.8 (5.6); 7.0 (8.0)	29.1
≥ 51 (n = 110)	5.4 (5.3); 4.0 (5.0)	6.3 (6.6); 4.0 (8.0)	25.5
**Gender**			
Male (n = 130)	6.2 (5.3); 5.0 (7.0)	6.7 (6.1); 5.0 (8.0)	28.5
Female (n = 945)	8.4 (5.7); 7.0 (8.0)	8.8 (6.3); 8.0 (10.0)	34.8
Identify in another way (n = 12)	10.2 (5.1); 10.5 (7.0)	11.3 (7.8); 13.0 (13.5)	50.0
**Veterinary profession**			
Veterinary surgeon (n = 682)	7.5 (5.5); 6.0 (8.0)	7.8 (6.1); 6.0 (8.0)	33.1
Veterinary nurse (n = 188)	9.9 (5.8); 9.0 (9.3)	10.7 (6.3); 10.0 (9.0)	42.0
Non-clinical (n = 136)	8.5 (6.1); 7.0 (9.0)	8.8 (6.4); 8.0 (10.0)	30.1
Technician (n = 58)	9.8 (5.3); 10.0 (7.8)	11.5 (6.8); 12.0 (11.5)	34.5
Other (n = 23)	7.2 (5.9); 7.0 (8.5)	6.9 (6.4); 4.0 (7.0)	21.7
Veterinary sector			
**Small animal first opinion**			
Yes (n = 760)	8.8 (5.8); 8.0 (9.0)	9.4 (6.4); 8.0 (10.0)	38.6
No (n = 327)	6.7 (5.2); 5.0 (7.0)	7.0 (6.0); 5.0 (8.0)	24.2
**Equine first opinion**			
Yes (n = 209)	6.6 (5.2); 5.0 (7.0)	6.8 (5.5); 5.0 (8.0)	24.4
No (n = 878)	8.6 (5.7); 8.0 (9.0)	9.0 (6.5); 8.0 (10.0)	36.6
**Farm first opinion**			
Yes (n = 125)	5.5 (4.7); 4.0 (6.0)	5.9 (5.9); 4.0 (7.0)	24.0
No (n = 962)	8.5 (5.7); 8.0 (9.0)	9.0 (6.4); 8.0 (9.0)	35.6
**Referral small animal**			
Yes (n = 113)	8.8 (6.1); 8.0 (8.0)	9.5 (7.3); 8.0 (10.0)	50.4
No (n = 974)	8.1 (5.6); 7.0 (8.8)	8.5 (6.2); 7.0 (10.0)	32.3
**Employment status**			
Employed (n = 1039)	8.2 (5.7); 7.0 (8.0)	8.6 (6.3); 7.0 (10.0)	34.7
Unemployed (n = 48)	8.0 (5.9); 7.0 (9.3)	9.7 (7.1); 8.0 (11.3)	22.9
**Relationship status**			
Relationship (n = 701)	7.8 (5.6); 6.0 (9.0)	7.9 (6.1); 7.0 (9.0)	29.2
No relationship (n = 232)	8.5 (5.7); 8.0 (8.0)	9.8 (6.7); 9.0 (11.0)	40.5
Dating (n = 154)	9.4 (6.0); 9.0 (9.8)	10.2 (6.8); 9.0 (9.0)	47.4
**Living with people**			
Yes (n = 889)	8.3 (5.7); 7.0 (8.0)	8.6 (6.4); 7.0 (10.0)	46.7
No (n = 198)	7.6 (5.4); 7.0 (8.0)	8.8 (6.3); 7.0 (9.8)	44.9
**Parent**			
Yes (n = 385)	7.2 (5.7); 6.0 (8.0)	7.3 (6.1); 6.0 (9.0)	23.9
No (n = 702)	8.8 (5.7); 8.0 (9.0)	9.3 (6.4); 8.0 (10.0)	39.9
**IPAQ physical activity category**			
High (n = 416)	8.3 (5.9); 7.0 (9.3)	9.5 (6.5); 8.0 (10.0)	31.3
Moderate (n = 446)	7.8 (5.4); 7.0 (8.0)	8.2 (6.0); 7.0 (9.0)	35.4
Low (n = 225)	8.7 (5.6); 8.0 (9.0)	9.7 (6.5); 8.0 (10.0)	37.3
**Country of residence**			
UK (n = 884)	8.0 (5.7); 7.0 (9.0)	8.3 (6.3); 7.0 (9.0)	33.1
North America (n = 104)	9.3 (5.6); 9.0 (7.0)	10.7 (6.0); 11.0 (7.3)	43.3
Other (n = 99)	8.5 (5.5); 8.0 (8.0)	9.4 (7.3); 7.0 (12.0)	34.3

Tables S2-4 include models assessing pet ownership and exercise on depression (Table S2), anxiety (Table S3) and suicidal ideation (Table S4)

Table S2 Model assessing the impact of pet ownership and exercise-related variables on depression (n = 1087)

Following model simplification, all significant variables remained except 'time spent working with animals' which lost significance, however 'physical activity category' gained significance, compared to the initial model with all factors included.

Characteristic	**Df**	**Sum square difference**	**Mean square difference**	**F value**	**P value**
Pet owner	1	8.69	8.69	7.19	0.007
Veterinary profession	4	58.48	14.61	12.09	<0.001
Country	2	11.21	5.61	4.63	0.010
Age	3	57.65	19.22	15.89	<0.001
Gender	2	12.35	6.17	5.11	0.006
Relationship status	2	13.52	6.76	5.59	0.004
Small animal first opinion	1	28.07	28.07	23.21	<0.001
Equine first opinion	1	5.43	5.43	4.49	0.034
Farm first opinion	1	25.53	25.53	21.11	<0.001
Running	1	18.81	18.81	15.56	<0.001
Walking	1	4.67	4.67	3.86	0.049
Physical activity category	2	8.67	4.34	3.59	0.028
Time spent sitting	1	5.99	5.99	4.95	0.026
Residuals	1064	1286.60	1.21		
**R^2 = 0.17** **Adjusted R^2 = 0.15**					

Table S3 The impact of pet ownership and exercise-related variables on anxiety (n = 1087)

Following model simplification 'being a parent' and 'physical activity category' became non-significant, after initially being significant in the model with all factors.

Characteristic	**df**	**Sum square difference**	**Mean square difference**	**F-value**	**P-value**
Veterinary Profession	4	39.61	9.90	8.99	<0.001
Age	3	68.91	22.97	20.85	<0.001
Gender	2	16.17	8.09	7.34	<0.001
Small animal first opinion sector	1	19.78	19.78	17.96	<0.001
Equine first opinion sector	1	5.23	5.23	4.74	0.030
Farm first opinion sector	1	23.79	23.78	21.59	<0.001
Proportion of time working with animals	1	6.35	6.35	5.77	0.016
Running	1	18.58	18.58	16.87	<0.001
Residuals	1072	1180.70	1.10		
**R^2 = 0.14** **Adjusted R^2 = 0.13**					

Table S4 Model assessing the impact of pet ownership and exercise-related variables on suicidal ideation (n = 1087)

There was no change in significant factors following model simplification

**Variable**	**df**	**Residual df**	**Residual deviance**	**Chi-squared**	**P value**
Age<br>*Relationship status*	3<br>2	1083<br>1081	1376.5<br>1364.2	20.34<br>12.24	< 0.001<br>0.002
Living with people<br>*Parent*<br>*Employment status*	1<br>1<br>1	1080<br>1079<br>1078	1360.2<br>1353.1<br>1347.0	3.99<br>7.13<br>6.14	0.046<br>0.008<br>0.013
Small animal first opinion	1	1077	1325.9	21.05	<0.001
Equine first opinion	1	1076	1322.0	3.91	0.048
Referral small animal	1	1075	1305.0	17.02	<0.001

Tables S5-7 include models assessing pet type on depression (Table S5), anxiety (Table S6) and suicidal ideation (Table S7)

Table S5 The impact of pet type on depression score (n = 941)

Following model simplification all significant factors remained except the amount of time spent sitting lost significance. Cat ownership and physical activity category gained significance compared to the original model with all factors included.

**Variable**	**df**	**Sum square difference**	**Mean square difference**	**F-value**	**P-value**
Cat ownership	1	5.23	5.23	4.37	0.037
Horse ownership	1	10.00	10.00	8.34	0.004
Other pets owned	1	7.09	7.09	5.92	0.015
Veterinary profession	4	60.74	15.18	12.66	<0.001
Age	3	51.31	17.10	14.26	<0.001
Gender	2	14.31	7.15	5.96	0.003
Relationship status	2	8.27	4.13	3.45	0.032
Small animal first opinion	1	14.49	14.49	12.08	<0.001
Farm first opinion	1	30.78	30.78	25.67	<0.001
Gym	1	8.12	8.12	6.77	0.009
Running	1	12.48	12.48	10.41	0.001
Physical activity category<br><br>***Residuals***	2<br><br>920	8.11<br><br>1103.33	4.05<br><br>0.034	3.38	0.034
**R^2 = 0.17**	**Adjusted R^2 = 0.16**				

Table S6 The impact of pet type on anxiety score (n = 941)

Following model simplification ‘living with people’ and ‘time spent working with animals’ gained significance.

Characteristic	**df**	**Sum square difference**	**Mean square difference**	**F-value**	**P-value**
Dog ownership	1	5.95	5.95	5.56	0.019
Horse ownership	1	5.99	5.99	5.60	0.018
Other pets owned	1	13.39	13.39	12.52	<0.001
Veterinary profession	4	40.06	10.01	9.36	<0.001
Age	3	60.55	20.18	18.87	<0.001
Gender	2	19.85	9.92	9.27	<0.001
Living with people	1	4.39	4.39	4.10	0.043
Parent	1	4.48	4.48	4.18	0.041
Small animal first opinion	1	12.86	12.85	12.02	<0.001
Farm first opinion	1	26.20	26.20	24.50	<0.001
Proportion of time working with animals	1	4.44	4.43	4.15	0.042
Reason for pet acquisition	2	7.48	3.74	3.50	0.031
Running	1	17.30	17.30	16.18	<0.001
Physical activity category	2	6.64	3.32	3.10	0.045
Residuals	918	981.83	1.07		
**R^2 = 0.19**	**Adjusted R^2 = 0.17**				

Table S7 The impact of pet type on suicidal ideation (n = 941)

All significant factors remained following model simplification and ‘farm animal first opinion’ gained significance

**Variable**	**df**	**Residual df**	**Residual deviance**	**Chi-squared**	**P-value**
Dog ownership	1	939	1208.0	5.04	0.025
Cat ownership	1	938	1197.5	10.52	0.001
Horse ownership	1	937	1193.2	4.32	0.038
Age	3	934	1177.5	15.69	0.001
Relationship status	2	952	1171.0	6.51	0.039
Parent	1	931	1163.8	7.18	0.007
Employment status	1	930	1157.7	6.09	0.014
Small animal first opinion	1	929	1146.3	11.35	<0.001
Farm first opinion	1	928	1141.9	4.39	0.036
Referral small animal	1	927	1127.8	14.18	<0.001

S1 Test of homogeneity of variance The homogeneity of variance across groups of all the categorical variables used in the regression models were tested with Levene's test. Most tests results were non-significant (p>0.05), which indicates that the groups are highly homogeneous. The variables below were significant, which could indicate heterogeneity between groups. Thus, we have calculated the standard deviation (SD) of each group of these variables and assessed whether the difference between SDs indicated heterogeneity between groups. As the SDs were very similar, we concluded that between-group comparisons could be made despite the difference in sample sizes.

- Relationship status
    - Depression model
        - Not in a relationship: SD = 5.68
        - Dating / Seeing someone on a regular basis: SD = 6.01
        - Married / Civil Partnership/ Cohabiting relationship: SD = 5.57
- Small animal first opinion - work sector
    - Anxiety model
        - No: SD = 5.22
        - Yes: SD = 5.76
    - Depression model:
        - No: SD = 5.95
        - Yes: SD = 6.42
- Equine first opinion - work sector
    - Anxiety model:
        - No: SD = 5.73
        - Yes: SD = 5.21
    - Depression model:
        - No: SD = 6.50
        - Yes: SD = 5.47
- Farm animals first opinion - work sector
    - Anxiety model:
        - No: SD = 5.71
        - Yes: SD = 4.71
    - Depression model:
        - No: SD = 6.38
        - Yes: SD = 5.59
- Referral equine - work sector
    - Anxiety model:
        - No: SD = 5.72
        - Yes: SD = 4.98
    - Depression model:
        - No: SD = 6.43
        - Yes: SD = 5.29
- Running
    - Anxiety model:
        - No: SD = 5.81
        - Yes: SD = 5.04
    - Depression model:
        - No: SD = 6.57
        - Yes: SD = 5.36
- Swimming
    - Depression model:
        - No: SD = 6.51
        - Yes: SD = 5.22
- Cycling
    - Depression model:
        - No: SD = 6.46
        - Yes: SD = 5.74
- Walking
    - Depression model:
        - No: SD = 6.74
        - Yes: SD = 6.20
- Not doing any exercise regularly:
    - Anxiety model:
        - No: SD = 5.59
        - Yes: SD = 6.22
    - Depression model:
        - No: SD = 6.21
        - Yes: SD = 7.20

Table S8 Parameter estimates for the impact of pet ownership and exercise-related variables on depression (n = 1,087)

The p-values here are not identical to the ones in Table S2 (type I ANOVA).

**Variables**	**Estimate**	**Standard error**	**t value**	**p-value**	
Intercept	2.73	0.24	11.17	0.000	***
**Pet owner (versus non-owner)**	0.34	0.10	3.29	0.001	**
**Profession**					
Veterinary surgeon (versus non-clinical)	-0.12	0.11	-1.01	0.311	
Technician (versus non-clinical)	0.18	0.20	0.88	0.382	
Veterinary nurse (versus non-clinical)	0.17	0.13	1.26	0.208	
Other profession (versus non-clinical)	-0.44	0.26	-1.69	0.091	
**Country**					
Country United Kingdom	-0.02	0.13	-0.16	0.877	
Country Other	-0.02	0.17	-0.10	0.920	
**Age**					
31-40 years old (versus 18-30)	-0.22	0.08	-2.58	0.010	*
41-50 years old (versus 18-30)	-0.31	0.11	-2.88	0.004	**
51 years and older (versus 18-30)	-0.70	0.13	-5.38	0.000	***
**Gender**					
Male (versus female)	-0.30	0.11	-2.78	0.006	**
In another way (versus female)	0.06	0.32	0.20	0.842	
**Relationship status**					
Married/Partnership/Cohabiting (versus dating)	-0.24	0.11	-2.23	0.026	*
Not in a relationship (versus dating)	0.04	0.12	0.31	0.758	
**Small animal first opinion sector (versus not)**	0.35	0.08	4.20	0.000	***
**Equine first opinion sector (versus not)**	-0.01	0.10	-0.07	0.943	
**Farm first opinion sector (versus not)**	-0.49	0.12	-4.24	0.000	***
**Running (versus not)**	-0.29	0.08	-3.57	0.000	***
**Walking (versus not)**	-0.13	0.08	-1.62	0.105	
**IPAQ category low (versus high)**	0.07	0.10	0.75	0.455	
**IPAQ category moderate (versus high)**	-0.13	0.08	-1.75	0.081	
**Time sitting**	0.02	0.01	2.23	0.026	*

Residual standard error: 1.10 on 1064 degrees of freedom. R^2 = 0.167. Adjusted R^2 = 0.150
*P<0.05, ** P<0.01, *** P<0.001

Table S9 Parameter estimates for the impact of pet ownership and exercise-related variables on anxiety (n = 1,087)

**Variables**	**Estimate**	**Standard error**	**t value**	**p-value**	
Intercept	2.96	0.13	22.64	0.000	***
**Profession**					
Veterinary surgeon (versus non-clinical)	-0.38	0.13	-2.89	0.004	**
Technician (versus non-clinical)	-0.16	0.19	-0.87	0.382	
Veterinary nurse (versus non-clinical)	-0.14	0.14	-0.97	0.331	
Other profession (versus non-clinical)	-0.49	0.24	-2.01	0.045	*
**Age**					
31-40 years old (versus 18-30)	-0.28	0.08	-3.73	0.000	***
41-50 years old (versus 18-30)	-0.31	0.10	-3.17	0.002	**
51 years and older (versus 18-30)	-0.84	0.12	-6.98	0.000	***
**Gender**					
Male (versus female)	-0.30	0.10	-2.98	0.003	**
In another way (versus female)	0.23	0.31	0.74	0.460	
**Small animal first opinion sector** (versus **not)**	0.22	0.08	2.74	0.006	**
**Equine first opinion sector** (versus **not)**	-0.06	0.10	-0.65	0.519	
**Farm first opinion sector (versus not)**	-0.48	0.11	-4.35	0.000	***
**Proportion of time working with animals**	0.08	0.03	2.32	0.021	*
**Running (versus not running)**	-0.31	0.08	-4.11	0.000	***

The p-values here are not identical to the ones in Table S3 (type I ANOVA).
Residual standard error: 1.05 on 1072 degrees of freedom. R^2 = 0.144. Adjusted R^2 = 0.132. *P<0.05, ** P<0.01, *** P<0.001

Table S10 Parameter estimates for the impact of pet ownership and exercise-related variables on suicidal ideation (n = 1,087) (The p-values here are not identical to the ones in Table S4 (type I ANOVA)

**Variables**	**Estimate**	**Standard error**	**z value**	**p-value**	
Intercept	-0.33	0.27	-1.21	0.226	
**Age**					
31-40 years old (versus 18-30)	-0.27	0.17	-1.60	0.109	
41-50 years old (versus 18-30)	-0.18	0.23	-0.79	0.431	
51 years and older (versus 18-30)	-0.27	0.28	-0.98	0.327	
**Relationship status**					
Married/Partnership/Cohabiting (versus dating)	-0.41	0.21	-1.98	0.048	*
Not in a relationship (versus dating)	-0.20	0.22	-0.91	0.364	
**Living with other people (versus not)**	-0.35	0.20	-1.80	0.072	
**Having children (versus not)**	-0.48	0.18	-2.73	0.006	**
**Unemployed (versus employed)**	-0.79	0.37	-2.16	0.031	*
**Small animal first opinion sector (versus not)**	0.76	0.17	4.37	0.000	***
**Equine first opinion sector (versus not)**	-0.21	0.20	-1.07	0.286	
**Referral small animal sector (versus not)**	0.90	0.22	4.13	0.000	***

Residual deviance: 1305 on 1075 degrees of freedom. AIC = 1329; *P<0.05, ** P<0.01, *** P<0.001

Table S11 Parameter estimates for the impact of pet type on depression (n = 941)

The p-values here are not identical to the ones in Table S5 (type I ANOVA).

**Variables**	**Estimate**	**Standard error**	**t value**	**p-value**	
Intercept	3.26	0.18	18.56	0.000	***
**Cat owner (versus not)**	0.05	0.07	0.64	0.522	
**Horse owner (versus not)**	-0.12	0.10	-1.22	0.222	
**Other type of pet (versus not)**	0.12	0.08	1.42	0.156	
**Profession**					
Veterinary surgeon (versus non-clinical)	-0.21	0.11	-1.83	0.068	
Technician (versus non-clinical)	0.15	0.19	0.81	0.418	
Veterinary nurse (versus non-clinical)	0.11	0.14	0.79	0.429	
Other profession (versus non-clinical)	-0.52	0.28	-1.88	0.060	
**Age**					
31-40 years old (versus 18-30)	-0.21	0.09	-2.28	0.023	*
41-50 years old (versus 18-30)	-0.33	0.11	-2.91	0.004	**
51 years and older (versus 18-30)	-0.76	0.14	-5.45	0.000	***
**Gender**					
Male (versus female)	-0.37	0.12	-3.18	0.002	**
In another way (versus female)	0.09	0.34	0.26	0.793	
**Relationship status**					
Married/Partnership/Cohabiting (versus dating)	-0.27	0.12	-2.28	0.023	*
Not in a relationship (versus dating)	-0.06	0.13	-0.48	0.633	
**Small animal first opinion sector (versus not)**	0.27	0.09	3.22	0.001	**
**Farm first opinion sector (versus not)**	-0.56	0.11	-4.94	0.000	***
Gym (versus not)	-0.20	0.09	-2.18	0.029	*
Running (versus not)	-0.28	0.09	-3.10	0.002	**
**IPAQ category low (versus high)**	0.11	0.11	1.00	0.317	
**IPAQ category moderate (versus high)**	-0.14	0.08	-1.64	0.102	

Residual standard error: 1.09 on 920 degrees of freedom. R^2 = 0.173. Adjusted R^2 = 0.155

*P<0.05, ** P<0.01, *** P<0.001

Table S12 Parameter estimates for the impact of pet type on anxiety (n = 941)

The p-values here are not identical to the ones in Table S6 (type I ANOVA).

**Variables**	**Estimate**	**Standard error**	**t value**	**p-value**	
Intercept	2.96	0.18	16.90	0.000	***
**Dog owner (versus not)**	-0.06	0.08	-0.79	0.428	
**Horse owner (versus not)**	-0.05	0.10	-0.51	0.612	
**Other type of pets (versus not)**	0.17	0.08	2.11	0.035	*
**Profession**					
Veterinary surgeon (versus non-clinical)	-0.37	0.14	-2.69	0.007	**
Technician (versus non-clinical)	-0.22	0.19	-1.14	0.256	
Veterinary nurse (versus non-clinical)	-0.14	0.15	-0.96	0.340	
Other profession (versus non-clinical)	-0.69	0.26	-2.65	0.008	**
**Age**					
31-40 years old (versus 18-30)	-0.19	0.09	-2.14	0.032	*
41-50 years old (versus 18-30)	-0.19	0.12	-1.68	0.094	
51 years and older (versus 18-30)	-0.78	0.14	-5.63	0.000	***
**Gender**					
Male (versus female)	-0.40	0.11	-3.62	0.000	***
In another way (versus female)	0.22	0.32	0.68	0.494	
**Living with other people (versus not)**	0.21	0.10	2.24	0.026	*
**Having children (versus not)**	-0.17	0.09	-2.03	0.043	*
**Small animal first opinion sector (versus not)**	0.22	0.08	2.69	0.007	**
**Farm first opinion sector (versus not)**	-0.53	0.11	-4.90	0.000	***
**Proportion of time working with animals**	0.07	0.04	2.02	0.044	*
**Reason for acquiring the pet**					
Pet acquired for exercise (versus companionship)	-0.27	0.11	-2.42	0.016	*
Pet acquired for other reason (versus companionship)	0.02	0.12	0.15	0.879	
**Running (versus not)**	-0.35	0.08	-4.19	0.000	***
**IPAQ category low (versus high)**	-0.06	0.10	-0.56	0.573	
**IPAQ category moderate (versus high)**	-0.19	0.08	-2.42	0.016	*

Residual standard error: 1.03 on 918 degrees of freedom. R^2 = 0.190. Adjusted R^2 = 0.170

*P<0.05, ** P<0.01, *** P<0.001

Table S13 Parameter estimates for the impact of pet type on suicidal ideation (n = 941)

The p-values here are not identical to the ones in Table S7 (type I ANOVA).

**Variables**	**Estimate**	**Standard error**	**z value**	**p-value**	
Intercept	-0.77	0.30	-2.54	0.011	*
**Dog owner (versus not)**	-0.06	0.17	-0.38	0.707	
**Cat owner (versus not)**	0.40	0.16	2.54	0.011	*
**Horse owner (versus not)**	-0.12	0.21	-0.57	0.568	
**Age**					
31-40 years old (versus 18-30)	-0.25	0.18	-1.37	0.172	
41-50 years old (versus 18-30)	-0.14	0.24	-0.58	0.565	
51 years and older (versus 18-30)	-0.26	0.30	-0.88	0.380	
**Relationship status**					
Married/Partnership/Cohabiting (versus dating)	-0.41	0.23	-1.78	0.074	
Not in a relationship (versus dating)	-0.07	0.25	-0.28	0.783	
**Having children (versus not)**	-0.50	0.19	-2.72	0.007	**
**Unemployed (versus employed)**	-0.74	0.39	-1.91	0.056	
**Small animal first opinion sector (versus not)**	0.75	0.19	4.03	0.000	***
**Farm first opinion sector (versus not)**	-0.46	0.24	-1.88	0.061	
**Referral small animal sector (versus not)**	0.88	0.23	3.77	0.000	***

Residual deviance: 1128 on 927 degrees of freedom. AIC = 1156

*P<0.05, ** P<0.01, *** P<0.001

SQ1 Questionnaire

1. Which role best describes your position within the veterinary profession?
- ☐ Veterinary surgeon
- ☐ Veterinary nurse
- ☐ Technician
- ☐ Non-clinical employee (e.g. practice manager/receptionist)
- ☐ Other

If you selected Other, please specify:

2. What is your place of residence?
- ☐ Africa
- ☐ Asia
- ☐ Australia
- ☐ Europe (other than the UK)
- ☐ New Zealand
- ☐ North America
- ☐ South America
- ☐ United Kingdom
- ☐ Other

If you selected Other, please specify:

3. What is your age?
- ☐ Less than 18 years old
- ☐ 18-30 years old
- ☐ 31-40 years old
- ☐ 41-50 years old
- ☐ 51-60 years old
- ☐ 61-70 years old
- ☐ 71 years or older

4. Which one of the following best describes your gender?
- ☐ Male
- ☐ Female
- ☐ In another way, please state:
- ☐ Prefer not to say

Please specify:

5. Relationship status?
- ☐ Married / Civil Partnership/ Cohabiting relationship
- ☐ Separated / Divorced / Widowed
- ☐ Dating / Seeing someone on a regular basis
- ☐ Single

6. Do you live with other people?
- ☐ Yes
- ☐ No

7. Do you have a child/children?
- ☐ Yes
- ☐ No

8. Current employment status?
- ☐ Full/part time employment
- ☐ Self-employed

- ☐ Unemployed
- ☐ Home maker
- ☐ Student
- ☐ Retired
- ☐ Other

9. Within what sector of the veterinary profession do you work? Multiple choices allowed
- ☐ Small animal first opinion
- ☐ Equine first opinion
- ☐ Farm first opinion
- ☐ Referral small animal
- ☐ Referral equine
- ☐ Industry
- ☐ Education, public sector, research
- ☐ Other

If you selected Other, please specify:

10. Approximately what proportion of your time at work is spent working with animals?
- ☐ 0%
- ☐ 25%
- ☐ 50%
- ☐ 75%
- ☐ 100%

11. Do you own a pet? (A pet is defined as any live animal, including invertebrates, which provides the owner with pleasure)
- ☐ Yes
- ☐ No

12. Please specify what pet/s you own. Multiple options allowed.
- ☐ Dog
- ☐ Cat
- ☐ Rabbit
- ☐ Reptile
- ☐ Horse
- ☐ Fish
- ☐ Rodent
- ☐ Invertebrates
- ☐ Other

If you selected Other, please specify:

13. What was your main reason for acquiring your pet/s?

- ☐ For companionship
- ☐ For leisure/walking/exercise/fitness
- ☐ For your children/other family member
- ☐ For work reasons/guarding a property
- ☐ For health reasons
- ☐ For disability assistance
- ☐ Other

14. Considering the tasks involved in looking after your pet/s on a day-to-day basis (e.g. feeding, giving water, cleaning), how often are these tasks performed by yourself?

- ☐ Never
- ☐ Almost never

- ☐ Sometimes
- ☐ Frequently
- ☐ Very frequently

15. Considering the provision of services for your pet/s (e.g., paying for food, health care, etc), how often are these services performed by you?

- ☐ Never
- ☐ Almost never
- ☐ Sometimes
- ☐ Frequently
- ☐ Very frequently

16. How often do you train your pet/s? (e.g. teach sit, roll over, recall etc)

- ☐ Never
- ☐ Almost never
- ☐ Sometimes
- ☐ Frequently
- ☐ Very frequently

17. How often do you interact with your pet/s? (e.g., being next to each other, touching, playing)

- ☐ Never
- ☐ Almost never
- ☐ Sometimes
- ☐ Frequently
- ☐ Very frequently

18. How often do you take your pet/s to work? (not for veterinary treatment)

- ☐ Never
- ☐ Almost never
- ☐ Sometimes
- ☐ Frequently
- ☐ Very frequently

19. How often does your pet/s present any behaviour problems (e.g., damaging furniture, house soiling, unwanted vocalising)?

- ☐ Never
- ☐ Almost never
- ☐ Sometimes
- ☐ Frequently
- ☐ Very frequently

20. How often do you socialise with other people because of your pet/s? (E.g., people approach you and talk to you about your pet/s).

- ☐ Never
- ☐ Almost never
- ☐ Sometimes
- ☐ Frequently
- ☐ Very frequently

21. What forms of exercise do you regularly participate in? Multiple choices allowed

- ☐ Gym
- ☐ Running
- ☐ Sports (football, rugby, tennis)

- ☐ Swimming
- ☐ Horse riding
- ☐ Cycling
- ☐ Walking/hiking
- ☐ Other
- ☐ None

If you selected Other please specify:

The following questions will ask you about the time you spent being physically active in the last 7 days. Please answer each question even if you do not consider yourself an active person. Consider activities you do at work, at home, and getting from place to place, as well as recreation, exercise, and sporting activities.

22. During the last 7 days, on how many days did you do vigorous physical activities like heavy lifting, digging, aerobics, or fast bicycling? Vigorous physical activities refer to activities that take hard physical effort and make you breathe much harder than normal. Think only about those physical activities that you did for at least 10 minutes at a time.

- ☐ No vigorous physical activity
- ☐ 1 day
- ☐ 2 days
- ☐ 3 days
- ☐ 4 days
- ☐ 5 days
- ☐ 6 days
- ☐ 7 days (everyday)

23. How much time did you spend doing vigorous physical activities on one of those days?

- ☐ 10 minutes
- ☐ 20 minutes
- ☐ 30 minutes
- ☐ 40 minutes
- ☐ 50 minutes
- ☐ 1 hour
- ☐ 1 hour 10 mins
- ☐ 1 hour 20 mins
- ☐ 1 hour 30 mins
- ☐ 1 hour 40 mins
- ☐ 1 hour 50 mins
- ☐ 2 hours
- ☐ More than 2 hours

24. During the last 7 days, on how many days did you do moderate physical activities like carrying light loads, bicycling at a regular pace, or tennis? Do NOT include walking. Moderate activities should take moderate physical effort and make you breathe someone harder than normal. Think only about those physical activities that you did for at least 10 minutes at a time.

- ☐ No moderate physical activity
- ☐ 1 day
- ☐ 2 days
- ☐ 3 days
- ☐ 4 days
- ☐ 5 days
- ☐ 6 days
- ☐ 7 days (everyday)

25. How much time did you spend doing moderate physical activities on one of those days?

- ☐ 10 minutes
- ☐ 20 minutes

- 30 minutes
- 40 minutes
- 50 minutes
- 1 hour
- 1 hour 10 minutes
- 1 hour 20 minutes
- 1 hour 30 minutes
- 1 hour 40 minutes
- 1 hour 50 minutes
- 2 hours
- More than 2 hours

26. During the last 7 days, on how many days did you walk for at least 10 minutes at a time? This can include at home, work, travelling from place to place or recreation and exercise.

- No walking activity
- 1 day
- 2 days
- 3 days
- 4 days
- 5 days
- 6 days
- 7 days (everyday)

27. How much time did you spend doing walking physical activities on one of those days?

- 10 minutes
- 20 minutes
- 30 minutes
- 40 minutes
- 50 minutes
- 1 hour
- 1 hour 10 minutes
- 1 hour 20 minutes
- 1 hour 30 minutes
- 1 hour 40 minutes
- 1 hour 50 minutes
- 2 hours
- More than 2 hours

28. During the last 7 days, how much time did you spend sitting on one weekday? This could include time sitting at a desk, visiting friends, reading, or watching television.

- 1 hour
- 2 hours
- 3 hours
- 4 hours
- 5 hours
- 6 hours
- 7 hours
- 8 hours
- 9 hours
- 10 hours
- 11 hours
- 12 hours
- 13 hours
- 14 hours
- 15 hours
- More than 15 hours

29. Over the last 2 weeks, how often were you bothered by the following problems?

	Not at all	Several days	More than half the days	Nearly every day
Feeling nervous, anxious or on edge				
Not being able to stop or control worrying				
Worrying too much about different things				
Trouble relaxing				
Being so restless that it is hard to sit still				
Becoming easily annoyed or irritable				
Feeling afraid as if something awful might happen				

30. Over the last 2 weeks, how often have you been bothered by any of the following problems?

	Not at all	Several days	More than half the days	Nearly every day
Little interest or pleasure in doing things				
Feeling down, depressed, or hopeless				
Trouble falling or staying asleep, or sleeping too much				
Feeling tired or having little energy				
Poor appetite or overeating				
Feeling bad about yourself or that you are a failure or have let yourself or your family down				
Trouble concentrating on things, such as reading the newspaper or watching television				
Moving or speaking so slowly that other people could have noticed? Or the opposite, being so fidgety or restless that you have been moving around a lot more than usual				
Thoughts that you would be better off dead or of hurting yourself in some way				

31. In the previous 12 months have you thought of taking your life, even if you would not really do it?

- ☐ Yes
- ☐ No
- ☐
